# Revisiting the Effect of Emotional Labor: A Multi-Level Investigation in Front-Line Service Teams

**DOI:** 10.3389/fpsyg.2020.570048

**Published:** 2020-10-09

**Authors:** Xin Zhao, Na Fu, Yseult Freeney, Patrick C. Flood

**Affiliations:** ^1^Business School, Northeastern University at Qinhuangdao, Qinhuangdao, China; ^2^Trinity Business School, Trinity College Dublin, The University of Dublin, Dublin, Ireland; ^3^DCU Business School, Dublin City University, Dublin, Ireland

**Keywords:** emotional labor, deep acting, surface acting, emotional exhaustion, task performance, customer loyalty

## Abstract

The main purpose of this study is to consider individuals in teams and to reexamine how emotional labor affects the performance of front-line service team and team members through emotional exhaustion. Multi-source data collection and a time-lagged research design was adopted to collect data from matched team members and customers nested in 82 front-line service teams in a large electronics provider based in China. The findings show that surface acting increases emotional exhaustion which reduces customer loyalty at the team level and individual task performance at the individual level, supporting a full mediation model. While, deep acting is not associated with emotional exhaustion, it is positively linked with team member’s task performance. This study provides evidence for the nested nature of emotional labor and exhaustion in teams.

## Introduction

Engaging enthusiastically in customer service delivery has become ever more critical for front-line service teams in order to create superior customer experience in today’s hyper-competitive market (Fernandes and Pinto, 2019). During the customer service delivery process, providing “service with a smile” is critical. However, literature shows that inauthentic “service with a smile” may be harmful for employee well-being and may reduce service quality (Johnson and Spector, 2007), a viewpoint which is strongly rooted in the emotional labor literature.

Emotional labor was originally defined by Arlie Hochschild as the “management of feeling to create a publicly facial and bodily display” (Hochschild, 1983: 7). For example, service employees’ effective emotional display, contributes significantly to effective customer services (Lee et al., 2019; Soderlund and Berg, 2019). Emotional labor is usually performed via two strategies, i.e., surface acting and deep acting (Grandey, 2003). Surface acting refers to situations where employees change their outward behavior to show the expected emotion which is not their real feeling (Zhang et al., 2018). Deep acting refers to those genuine positive feelings when employees display smiles to customers (Gabriel et al., 2015). Given the importance of employees’ emotional display toward customers, research has revealed the impact of surface acting and deep acting on employee wellbeing and performance (Humphrey et al., 2015). Despite the progress made to understand emotional labor, there are still several important but unanswered questions.

First of all, the underlying mechanisms as to how emotional labor influences individual and team outcomes are not yet well understood. Existing studies have focused on individual employees in the service context (e.g., Chi and Chen, 2019; Jeong et al., 2019). In practice, the majority of employees work in teams to deliver service to customers. Almost 75% of organizations have adopted the team-based service model (Cummings, 2007). The service team plays an important role in customer relationship management, as teams provide a one-stop service and provide a solution center for the customers. It is an effective approach to understanding customers and building and maintaining deeper customer relationships (Deeter-Schmelz and Ramsey, 2003; Auh et al., 2014). In front-line service teams, there are many interactions between the team and customers, as well as among team members during the service delivery process. Prior studies on emotional labor have either created simulated individual-based service environments through experimental work or the sample used are a heterogeneous mix of various individual employee’s service roles (e.g., Groth et al., 2009), where service employees combine customer service and other types of customer interaction roles. The context of such teams which has been omitted from existing research needs to be investigated. Second, in terms of research design, existing research on the effects of emotional labor mainly adopts the single-source and cross-sectional design approach (Grandey, 2000), whereas multi-source and longitudinal research design is more recently encouraged (Choi et al., 2019).

To address these unanswered questions, this study selected Conservation of Resources (COR) theory as the theoretical background for our model. Drawing on COR, it theorizes the indirect link between emotional labor and individual and team outcomes via the mediating role of emotional exhaustion. This model is tested on front-line service teams in our study. This study adopts a multi-source data collection and time-lagged research design where data were collected from matched team members and customers nested in 82 service teams in a large electronics provider based in China. Based on the nested nature of the data, multi-level modeling was conducted. Findings and implications are discussed.

Our study makes both theoretical and managerial contributions to both the emotional labor and service team management literatures. Firstly, this study puts individuals back within their team context using a multi-level study which enriches emotional labor research. This study considers individuals as nested within teams, and in so doing, investigates the nature of emotional labor and exhaustion in teams. Secondly, by adopting multi-source data collection, our study contributes to the emotional labor literature by including the role of customers in the value chain. The multi-source data collection and time-lagged research design thus extends emotional labor research.

## Literature Review and Hypotheses Development

### Emotional Labor and Individual Exhaustion in Service Teams

The displayed emotions by employees who have direct interactions with customers have become an important marketing practice and a necessary job requirement. Both managers and employees view “service with a smile” as a precondition for high performance and a prerequisite for maintaining long-term customer relationships (Woo and Chan, 2020). Emotional labor, as the self-regulation process used by employees to display emotions in line with organizational display rules, becomes a core aspect of customer-contact employees’ daily roles (Grandey, 2000). Such emotion display by employees is labeled as emotional labor (Hochschild, 1983).

Emotional labor is normally performed through two strategies, surface acting and deep acting. The deep actors display authentic emotion, while surface actors change their outward behavior to show the expected emotion which lacks authenticity (Grandey, 2003). When employees display smiles to customers, employees engaging with deep acting would be genuinely happy to provide service to their customers. Employees engaging with surface acting would not be genuinely happy to provide service to their customers but still display the smiles usually required by the organizations. As a result of this distinction, a large number of studies have investigated the inverse impact of surface acting and deep acting toward individuals’ outcomes. For instance, surface acting gives rise to jobs stress (Choi et al., 2019) and emotional exhaustion (Zapf, 2002; Grandey, 2003), while, deep acting is negatively associated with job stress (Choi et al., 2019), and emotional exhaustion (Allen et al., 2014). Deep acting is also positively associated with well-being (Yao et al., 2019), and contributes to building good customer relationships, while surface acting has the opposite effect (Chi and Chen, 2019).

The above-mentioned studies almost exclusively rely on individual-based employee working models where data are collected from individual respondents. In reality, employees mostly work in teams (Cummings, 2007). Team-working environments entail interpersonal relationships and may require compliance with organizational display rules. Such a distinct context may influence the individual employees’ behavior and attitude, and finally change or fine-tune the mechanism of emotional labor. The difficulty to get access to teams has prevented researchers to understand the phenomena at the level it should be examined. As a result, the study of emotional labor in a service team context is quite sparse but needed.

Although situated in the team context, the well-established relationships between individual employee emotional labor and their well-being as well as task performance are expected to still hold up. Specifically, in order to follow organizational display rules, the surface actors suppress their true feelings and simply fake appropriate displays (Grandey, 2003). This emotional dissonance is unpleasant (Indregard et al., 2018). Based on COR theory, employees strive to obtain, maintain and create resources that they prize (Hobfoll, 1989). Moreover, unpleasant feelings arise from the loss of personal resources, which make individuals prone to emotional exhaustion (Wright and Bonett, 1997; Prapanjaroensin et al., 2017). Additionally, in service teams, besides interacting with customers, employees also interact frequently with other team members. Almost two-thirds of employee-employee interactions involve emotional regulation (Mann, 1999). On the basis of COR theory, if employees need frequent face-to-face interaction, they will feel emotional and physical depletion, a lack of energy, and even extreme tiredness (Lee and Ok, 2014). Therefore, for a surface actor, these emotional regulation demands further burden employees (Scherer et al., 2020; Yeung and Wong, 2020). Thus, we echo those findings which demonstrate a strong positive relationship between surface acting and emotional exhaustion in service teams.

However, in service teams, there are also deep actors who authentically display their emotions (Grandey, 2000). This kind of emotional display not only aligns with their true feelings but also activates emotional resources arising from their sincere customer service. Therefore, deep acting alleviates the employee’s emotional exhaustion.

Based on the foregoing discussion, we propose the following hypothesis:

H1a. Surface acting is positively related to individual emotional exhaustion in service teams.
H1b. Deep acting is negatively related to emotional exhaustion (1b) in service teams.

### The Mediating Role of Employee Exhaustion in Service Teams

Employee exhaustion has been found to be negatively linked to individual performance (e.g., Cuyper et al., 2014). When team members feel exhausted, they lack energy and their emotional resources are depleted (Soderlund and Berg, 2019). Accordingly, the exhausted employee cannot provide the expected customer service. As per COR theory, if the exhausted employees’ work demands exceed resources, task performance will be reduced (Cuyper et al., 2014).

In this study, we argue that emotional labor influences employee exhaustion where surface acting increases emotional exhaustion and deep acting reduces emotional exhaustion in the service team context. Employee exhaustion leads to lower performance. Therefore, we anticipate that emotional labor influences individual performance via the mediating role of employee exhaustion.

*H2. Emotional exhaustion mediates the link between emotional labor* (surface acting *2a;* deep acting *2b) and individual task performance in service teams.*

When individual team members are exhausted, the quality of service they provide to their customer is reduced. Customers will be less likely to stay with this organization. The logic of these prediction is based on the following reasons.

Customer loyalty as a service team’s performance indicator, refers to a customer’s intention to return (Stein and Ramaseshan, 2019), which depends on the service teams’ attitude and behaviors. The service team employees’ positive interaction with customers is beneficial to build customer loyalty (Waseem et al., 2018). However, exhausted service team employees experience reduced wellbeing associated with resource depletion. According to COR theory, employees will strive to save resources to cope with such adverse experiences. Consequently, exhausted employees choose to distance themselves from customers (Lee and Ok, 2014), and behave negatively during the interaction process, dissatisfying customers emotional service expectation, and hindering effective customer service (Karatepe et al., 2009). As the “customer is king” philosophy rules in many service environments, customers demand enthusiastic service. What is more, the exhausted service team employees who provide lower level service quality (Karatepe et al., 2009) decrease long-term customer relationships directly (Van Jaarsveld et al., 2010). Furthermore, due to the contagious nature of emotions (Woo and Chan, 2020), customers are likely to exit from a transaction with an emotionally exhausted service team. Meanwhile, customers will associate more negative emotions and experiences with that transaction. In line with the predictions of operant conditioning (Skinner, 1992), those customers are less likely to repeat the transaction through future service due to the negative associations with the first experience. Under the law of operant conditioning, if a behavior produces a particular consequence, it is more likely to occur again under similar circumstances. The behavior is reinforced by its consequences, and the consequences having this effect are named as reinforcers. Simintiras and Cadogan (1996) adopted operant conditioning theory to explain salesperson-customer interactions and claimed that the reinforcements can be either positive or negative. One of the three reinforcements in employee-customers interaction is punishment, “by providing an aversive consequence after a particular response, works in an opposite direction to reinforcement, leading to extinction or the reduction in the rate of emission of a behavior” (Simintiras and Cadogan, 1996, p.61). Therefore, if customers experience the services from an exhausted service team, it is likely to reduce the probability of future patronage, and consequently lower customer loyalty. Hence, front-line service team members’ emotional exhaustion will negatively influence long-term customer relationship.

Based on the aforementioned analysis, we propose that surface actors can experience aggregated emotional exhaustion, which in turn lowers customer loyalty, while deep actors could avoid or alleviate emotional exhaustion, thus boosting customer loyalty.

*H3. Emotional exhaustion mediates the link between emotional labor* (surface acting *3a;* deep acting *3b) and customer loyalty in front-line service teams.*

**FIGURE 1:**
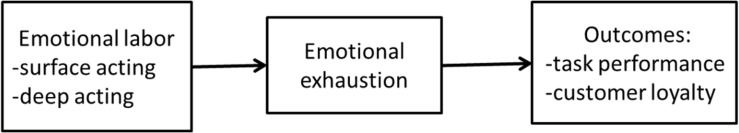
Theoretical model.

Figure 1 illustrates the theoretical model of this study.

## Research Methodology

### Sampling Procedure

We collected data from Chinese front-line service team and customers using hard-copy survey in a large electronics appliance provider. Based on the support from the senior HR manager in the northeastern region, we got access to 89 service teams across 12 stores in a large north-eastern city with over eight million people. We designed a coding system based on the services teams’ personnel information provided by the HR department. We put coded survey in envelopes and the sample organizations’ store managers help us to distribute them in their store. Respondents returned their complete surveys in an enclosed envelope without any identifiable information to their store managers. Using the question in the survey on which product function they were working, the researchers matched the data. The number of respondents from service teams ranged from 3 to 6 (mean = 4.18, SD = 0.52), and the response rates ranged from 67 to 100% (mean = 96%, SD = 9%).

To reduce common method bias (Podsakoff et al., 2003), data on the dependent variable – customer loyalty were collected from customers directly 1 week after collecting service team members’ data. Customers were given a pre-coded survey by the sales team after interacting with team members. Customers returned their surveys to the sales teams in a sealed envelope. In order to get a sufficient sample, we targeted 15 customer surveys for each sales team and 1,335 customer surveys were randomly distributed in the stores.

### Sample Profile

After deleting incomplete and invalid responses, the sample consisted of 264 employee responses (72%) nested in 88 service teams. Among the employees, 64% were female; 17% were under 25 years old, 74% were between 25 and 40 years old, and 9% were between 41 and 54 years old; 34% had secondary school education, 53% had a Diploma, and 14% had a Bachelor’s degree. Employee’s average work tenure was 4.01 years (SD = 3.35).

On the customer side, 1035 responses were received (78%). After deleting incomplete and invalid responses, there were 1003 customer responses (75%) nested in 88 service teams. Among the customers, 52% were female; 18% were under 25 years old, 52% were between 25 and 40 years old, 24% were between 41 and 54 years old, and 6% were 55 years old and above.

After matching the team members and customers, 82 service teams had both team member and customer data and were used as the final sample in the analysis.

### Measures

All constructs were adopted from published studies. They were measured on a five-point Likert Scale (from 1 = strongly disagree to 5 = strongly agree). We are aware of the debate on either five- or seven-point scale (Cummins and Gullone, 2000), or even more point scales (e.g., 10 in Dawes, 2008) used to capture respondents’ opinions and attitudes. A number of studies have changed the original seven-point to five-point either for simplicity or consistency with other scales in the survey. For example, emotional exhaustion was measured based on a five-point Likert scale in Conway et al. (2016) and Kilroy et al. (2020). Based on the fact that five-point scales seem less confusing and can help to increase response rate (Babakus and Mangold, 1992; Hayes, 2017), we adopted the five-point scale.

#### Emotional Labor

For emotional labor, we used Brotheridge and Lee’s (2003) measure. This six-item measure has multi-item subscales corresponding to two dimensions: surface acting and deep acting. Example items include: “I pretend to have emotions that I do not really have” (surface acting), and “I try to actually experience the emotions that I must show” (deep acting). As emotional labor is commonly operationalized as two sub-constructs – surface acting and deep acting, which has been used in a number of studies (e.g., Allen et al., 2014), we treated surface acting and deep acting separately. The reliability alpha coefficients for these two scales were 0.84 for surface acting and 0.82 for deep acting. Confirmatory factor analysis was conducted along with other employee measures including emotional exhaustion and individual job performance in order to examine the convergent and discrimination validity which is presented in the “Factor Analyses, Common Method Bias and Team Membership” section.

#### Emotional Exhaustion

We used a short version from Maslach and Jackson (1981) and asked team members to indicate the frequency (1 = never to 5 = always) of their feelings at work such as “I feel emotionally drained from my work” and “I feel used up at the end of the workday.” The reliability alpha coefficient was 0.91.

#### Individual Task Performance

For individual task performance, we used Peng et al. (2014) measure which was adapted from Williams and Anderson (1991). Example items include: “I perform all tasks that are expected of me,” and “I fulfill responsibilities specified in my job description.” The reliability coefficient alpha was 0.86.

#### Customer Loyalty

We adopted five items from Liao and Chuang (2004) which focuses on the customer loyalty to the service teams. Example items include: “I will recommend this department to others,” and “I am sure that I will not visit this department again” (reversed). The reliability of this measure is 0.75.

#### Control Variables

As team size could influence the members’ service experience, it is, therefore, controlled. The natural log algorithm is used. Another control variable is employee gender, which has been found to be correlated with employees’ emotional labor and outcomes (e.g., Johnson and Spector, 2007).

### Factor Analyses, Common Method Bias and Team Membership

To examine the convergent and discriminant validity of the scales in this study, we conducted confirmatory factor analysis for employee data. The fit indices for the CFA indicated very good model-fit (*χ^2^*/*df* = 97.35/59 = 1.65, *p* < 0.01; CFI = 0.98, RMSEA = 0.05, SRMR = 0.05). The factor loadings were all above 0.70 and significant at 0.001 level.

As the data on emotional labor, emotional exhaustion and task performance were collected from the same source, common method bias may exist (Podsakoff et al., 2003). In order to address the potential concern on common method bias, we have followed a number of recommendations by Podsakoff et al. (2003) and Podsakoff et al. (2012) in both the research design and analysis stages. During the research design stage, the survey has been tested, revised and retested a number of times among a group of experienced researchers, the senior HR manager and employees in the sample organization. During the distribution stage, we clarified the confidentiality of the survey and data in the cover letter and used enclosed envelopes without any identifiable information. Finally, during the data analysis stage, we carried out a series of CFA to compare them with the four-factor model in this study. Table 1 presents the results. These results show that the four-factor model in this study is the best fit model. In addition, the current research design of the multi-source data collection and time-lagged research design (team member survey returned within 1-week time and customer survey returned within 2-month time) helps to reduce the common method bias.

**TABLE 1:** Measurement model analysis results.

Models	*χ^2^/df*	*CFI*	*RMSEA*	*SRMR*	*χ^2^ difference*	*df difference*
Full measurement model	97.35/59	0.98	0.05	0.05		
Model A^*a*^	385.41/62	0.83	0.14	0.10	288.06***	3
Model B^*b*^	371.73/62	0.88	0.10	0.09	274.38***	3
Model C^*c*^ (Harman’s Single Factor Test)	1196.97/65	0.39	0.26	0.21	1099.62***	6

*N* = 263, ***p < 0.001; χ^2^, chi-square discrepancy; df, degrees of freedom; CFI, Comparative Fit Index; RMSEA, Root Mean Square Error of Approximation; SRMR, Standardized Root Mean Square Residual. In all measurement models, error terms were free to covary to improve fit and help reduce bias in the estimated parameter values. All models are compared to the full measurement model. ^*a*^ = emotional labor (surface acting) and emotional exhaustion combined into a single factor. ^*b*^ = emotional labor (deep acting) and task performance combined into a single factor. ^*c*^ = All factors combined into a single factor.

As the data are nested in teams, we firstly assess the nested nature of our employee and customer data in order to decide if multilevel or single level analysis is more appropriate. Using McGraw and Wong (1996) formula with a one-way random-effects analysis of variance, the ICC (1) values were as follows: emotional labor-surface acting (0.24), emotional labor-deep acting (0.21), emotional exhaustion (0.25), and individual task performance (0.20). These values are higher than James’ threshold (0.12), which confirms the nested structure of the data, where team membership does substantially influence participants’ responses. For customer loyalty, ICC1 = 0.29. Another indicator, ICC2, provides an overall estimate of the reliability of team means, with values equal to or above 0.70 being acceptable. The ICC2 value for customer loyalty is 0.83. As customers’ responses are based on the departments/teams, also based on the high ICCs, we aggregate customers’ responses into teams.

Overall, these results indicate that our data are nested at a higher level. The adoption of a multi-level approach is therefore critical, and we tested our hypotheses using multi-level modeling in Mplus. In the multi-level model, team members’ ratings on emotional labor, emotional exhaustion and task performance were treated at the individual level as these variables are individually based (within level). Customers’ ratings of their loyalty were analyzed at the team level (between level).

## Results

Table 2 shows the descriptive statistics on means, standard deviations, and correlations between variables.

**TABLE 2:** Descriptive statistics and correlations of study variables.

Variables	Mean	SD	Skewness	Kurtosis	1	2	3	4	5	6	7	8	9
**Between level (Level 2)**													
1. Customer loyalty	3.83	0.42	–0.01	–0.02									
2. Team size	1.42	0.10	1.93	5.67	00.01								
**Within level (Level 1)**													
3. Individual task performance	4.14	0.57	–0.31	0.69	0.18**	0.14*							
4. Emotional exhaustion	2.82	1.04	0.25	–0.56	−0.35**	–0.07	–0.11						
5. Emotional labor - surface acting	2.73	0.92	0.42	–0.24	−0.16*	–0.02	–0.03	0.38**					
6. Emotional labor - deep acting	3.86	0.71	–0.41	0.42	–0.03	0.05	0.54**	0.13*	0.15*				
7. Gender	0.36	0.48	0.60	–1.65	0.03	0.21**	0.17**	0.04	–0.02	0.04			
8. Education	1.8	0.68	0.08	–0.47	0.10	0.14*	0.11	–0.10	−0.14*	0.01	0.19**		
9. Tenure in the team (months)	22.76	28.35	3.26	16.58	0.01	0.01	–0.05	0.06	0.09	0.04	−0.13*	–0.07	
10. Workload	52.57	19.31	–0.87	0.70	−0.27**	–0.12	0.00	0.18**	0.14*	0.02	0.06	0.12	0.12

The Level 2 variables were disaggregated before calculating within-level correlations. *N* = 229 at individual level (Listwise); *N* = 82 at team level (Listwise). *p < 0.05 (Two-tailed) **p < 0.01.

**FIGURE 2:**
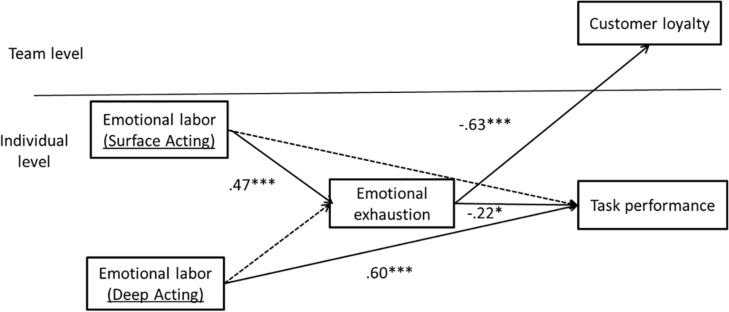
Two-level path analysis results^*a*^. *N* = 229, *R*^2^ emotional exhaustion = 0.28. *R*^2^ task performance = 0.37. *R*^2^ customer loyalty = 0.55. Standardized coefficients are presented. a: Control variables are not presented. —-> means that there is significant relationship between them *p < 0.05, ***p < 0.001.

Hypothesis 1 proposed that emotional labor has an impact on emotional exhaustion, where surface acting would be positively related to emotional exhaustion (1a) and deep acting would be negatively related to emotional exhaustion (1b). As shown in Figure 2, only surface acting was found to be negatively linked to emotional exhaustion (β = 0.47, *p* < 0.001) while deep acting was not found to be linked to emotional exhaustion (β = 0.11, n.s.). Therefore, Hypothesis 1 was partially supported and only H1a was supported.

Hypothesis 2 proposed that emotional exhaustion would mediate the link between emotional labor (surface acting – 3a; deep acting – 3b) and individual task performance. According to Hayes (2017), the mediation or indirect effect is based on the effect of the independent variable (emotional labor) on the mediator (emotional exhaustion) as well as the effect of the mediator (emotional exhaustion) on the dependent variable (task performance). The first condition was met by the supported link between surface acting and emotional exhaustion (supported Hypothesis 1a). The second condition was met by the significant relationship between emotional exhaustion and individual task performance (β = −0.22, *p* < 0.05). We calculated the indirect effect of surface acting and task performance via emotional exhaustion in Mplus (*z* = −0.07, *p* < 0.05), supporting the mediation model. The link between deep acting and emotional exhaustion is not significant (unsupported hypothesis 1b), suggesting no support for the mediation model. The indirect effect of deep acting and task performance via emotional exhaustion was not significant (*z* = −0.02, n.s.). Therefore, Hypothesis 2 was partially supported and only H2a was supported.

Hypothesis 3 proposed that emotional exhaustion would mediate the link between emotional labor (surface acting – 4a; deep acting – 4b) and customer loyalty. Similar to the above mediation analysis, the first condition was met by the supported link between surface acting and emotional exhaustion (supported Hypothesis 1a). The second condition was met by the significant relationship between emotional exhaustion and customer loyalty (β = −0.63, *p* < 0.001). We then calculated the indirect effect of surface acting and customer loyalty to the team via emotional exhaustion in Mplus (*z* = −0.26, *p* < 0.05). The indirect effect of deep acting on customer loyalty via emotional exhaustion was not significant (*z* = −0.08, n.s.). Therefore, hypothesis 3 was partially supported where only 3a was supported.

As shown in Figure 2, we additionally found a significant relationship between deep acting and individual task performance (β = 0.60, *p* < 0.001). The relationships between deep acting and customer loyalty as well as between surface acting with individual task performance and customer loyalty were not significant and not shown in the Figure 2.

## Discussion

Displaying positive emotions, or service with a smile, has been a consistent mantra for front-line service teams. Organizations often have an explicit selecting criterion in “hiring smiley faces” for the customer service roles (Goldberg and Grandey, 2007: page 312). It is noted that not all smiles are genuine and the consequences vary accordingly. Using data collected from service team members and their customers, the results from multi-level analysis reveal that emotional labor has an impact on team member exhaustion where surface acting increases exhaustion and deep acting does not affect exhaustion. Individual team member exhaustion is negatively linked with individual performance and team performance which is operationalized by customer loyalty. In addition, deep acting is positively linked with individual performance. These findings contribute to the emotional labor literature by testing the nested nature of emotional labor in teams using multi-source data and time-lagged research design and bear important managerial implications.

### Scholarly Implications

The first contribution of this study is to reveal the underlying mechanism between emotional labor and individual and team outcomes by theorizing and testing the mediating role of emotional exhaustion. There has been extensive research exploring the antecedents of emotional labor considering individual, task-related and organizational factors, such as gender (Grandey, 2000), social support (Chu, 2002). There are also numerous studies looking the direct impact of emotional labor on the individual and organizational outcomes. For example, emotional labor was found to be linked with individual job satisfaction (Li and Wang, 2016; Cheung et al., 2018) and organizational wellbeing (Grandey, 2000). However, the indirect link between emotional labor and individual and team outcomes still needs to be further studied (Choi et al., 2019). This study found the individual exhaustion mediated the link between emotional labor in the service team context. Specifically, surface acting strongly decreases team employee task performance via increased emotional exhaustion. The direct relationship between surface acting and task performance was tested in some other studies, such as Hülsheger and Schewe (2011) which reported a correlation of −0.114. However, our finding shows the indirect effect between them via exhaustion which is stronger than the direct effect. Furthermore, in some prior research, scholars found a direct or indirect relationship between emotional labor and other individual performance indicators, such as the indirect relationship between surface acting and general job performance via affective delivery (Goodwin et al., 2011) and adaptive selling behavior (Wang et al., 2016). Another example is the direct relationship between emotional labor and individual service performance (Tang et al., 2017; Van Gelderen et al., 2017). In such cases, our findings extend and broaden our understanding of the linkage between the emotional labor and individual performance. Furthermore, this study theorized and tested the model in the context of front-line service teams which has been commonly applied in organizations during the service process. Our approach considers individual responses within their team context and analyses the relationships within teams using a multi-level modeling method. In most previous studies, the difficulty of accessing teams has prevented researchers from understanding the phenomena at team level, which better reflects the reality of retail environments.

The findings of the negative impact of surface acting on individual wellbeing are consistent with existing research on emotional labor, such as those of Goldberg and Grandey (2007). When employees engage in surface acting, they suppress their true emotion, deplete their emotional and physical resources, and consequently experience emotional exhaustion (Grandey, 2003). Importantly, compared to previous findings, the effect size of our findings on the link between surface acting and emotional exhaustion is stronger than findings in individual-based selling models. Specifically, the correlation in our research is 0.47 which is higher than typically reported in individual-based selling model, such as Brotheridge and Grandey (2002) (*r* = 0.20), Goldberg and Grandey (2007) (*r* = 0.31), and Grandey (2003) (*r* = 0.33). Thus, this study provides strong support for the team level effects of emotional labor.

The findings on the non-significant impact of deep acting on employee wellbeing are inconsistent with prior studies, such as the study by Allen et al. (2014). Based on the literature, an explanation for the null relationship between deep acting and exhaustion is that deep acting does not only deplete but also replenishes resources (Hülsheger and Schewe, 2011). Although, most researchers claim that deep acting requires less mental resources than surface acting (e.g., Grandey, 2003; Totterdell and Holman, 2003), it still consumes employees’ cognitive and motivational resources as it requires regulation of emotions (Baumeister et al., 1998; Goldberg and Grandey, 2007). Therefore, the null relationship results from offsetting the positive effects, such as the positive emotions (Allen et al., 2014) and personal accomplishment (Brotheridge and Lee, 2002) arising from deep acting, and the negative effects, such as the effort involved in deep acting and the potential to be alienated from one’s true feelings (Hochschild, 1983).

The findings on the negative effect of surface acting show that it strongly decreases team member’s task performance via the increased emotional exhaustion. As mentioned before, the direct relationship between surface acting and task performance was tested in some other studies, such as the study by Cuyper et al. (2014). However, our findings not only demonstrate the indirect effect between them via exhaustion, but also show the detrimental effect of surface acting on task performance is more serious in team contexts. This finding enriches the current understanding of the relationship between surface acting and task performance. More importantly, our findings demonstrate the indirect effect of surface acting on customer loyalty, via emotional exhaustion. The existing studies have not explored the indirect relationship between surface acting and customer loyalty (e.g., Groth et al., 2009). Our study undoubtedly confirms the indirect relationship between surface acting and customer loyalty via exhaustion. This finding implies that surface acting is strongly associated with exhaustion, which indirectly reduces customer loyalty. Therefore, forcing retail sales-team employees to engage in inauthentic smiling is pointless.

The findings on the positive impact of deep acting on individual performance are consistent with existing research (Cuyper et al., 2014). This implies that the positive effects of deep acting would provide employee motivation and confidence at work and encourages them to perform better. We did not find a significant relationship between deep acting and customer loyalty at team level. This result needs further research to explore why and how deep acting influences customer loyalty.

Finally, as mentioned before, our findings are based on 82 front-line service teams with matched team members and customers from an actual field setting. This multi-source data collection and time-lagged research design contributes to emotional labor research in that we test immediate emotional labor behaviors and their effects as they are experienced by front-line service teams and their customers. Most previous studies on the effects of emotional labor rely solely on single source data from employees (e.g., Choi et al., 2019), which increases the problems arising from common-variance method, or on student samples, which are inherently limited as they lack external validity (e.g., Hennig-Thurau et al., 2006). The time-lagged research design help to reduce the common bias.

### Managerial Implications

Our study challenges the traditional management practice assumptions, particularly in the front-line service team context, with findings that problems may arise from mandatory emotional display. Front-line service teams, as the outward face of the organization, help to maintain the organizational image through their emotional displays (Soderlund and Berg, 2019). Our findings suggest that managers as well as front-line service team employees should more carefully manage emotional displays. Our findings showed that surface actors are more easily exhausted, which potentially reduces individual performance and customer loyalty. These negative effects are harmful for team effectiveness. Therefore, managers should carefully manage surface acting. As retail service teams engaged in high pressure jobs, managers should create positive working environments, using context enhancement such as pleasant music to adjust employee emotion, to encourage natural smiling behavior and to nurture deep acting. Evidence shows that “sales associates at an upscale clothing store might engage in deep acting by listening to pleasant music as they drive to work, chatting amiably with their co-workers once they arrive, and getting excited about fashion trends and the latest line of clothing they are carrying” (Humphrey et al., 2015). Similar methods should be adopted in sales teams in order to alleviate employees’ exhausted emotions.

Furthermore, when team employees cannot provide deep acting to customers, managers should be aware of fake emotional displays and encourage employees to engage in deep acting. For instance, team leaders should carefully observe and be sensitive to team members’ emotions and the need for sales associate to have breaks and not overlook. As negative events increase the possibility of surface acting (Grandey, 2000), employees should be temporarily redeployed to non-customer facing activities following stressful or negative events. Meanwhile, the team leader should encourage teamwork and team member collaboration in order to balance deep acting across team members.

On the other hand, our findings confirm the positive effect of deep acting on individual performance. The deep actor outperforms on individual tasks. Front-line managers could use deep acting in service as a criterion in selection and performance assessment. Specific training for front-line service teams is also needed to fostering effective deep acting approaches. Worker wellbeing is the platform for deep acting and managers and organizations need to recognize this fact and act upon.

### Limitations and Future Direction

Despite the theoretical and practical implications for the emotional labor and service team management literature, our study also has limitations that must be taken into account in future research. First, we consider the effect of two emotional labor strategies (surface acting and deep acting) on the performance from individual level. How team-level emotional labor influences team performance should be investigated in future studies.

Second, our findings suggest that there is no indirect effect of deep acting on customer loyalty via exhaustion. However, the emotional labor literature shows those employees’ deep acting benefits customer relationships. How deep acting benefits customer loyalty via other variables in team-based selling model is a fruitful area for investigation and provides good research question for both the emotional labor literature and the service team management literature.

## Conclusion

This study reveals that emotional labor influences individual and team outcomes indirectly. It situates the emotional labor research into the service teams where a multi-level analysis was conducted to investigate the impact of emotional labor on exhaustion and performance at individual level and customer loyalty at team level. The investigation of the indirect impact of emotional labor on individual task performance and customer loyalty as well as the positioning of emotional labor research in the multi-level context has extended our understanding of how emotional labor influences individual and teams. This study identifies the mediating role of exhaustion in explaining how and why emotional labor affects individual and team performance. Future research is called to examine the conditions under which emotional labor could influence individuals and teams. In doing so, organizations will be able to implement interventions to reduce the harmful effect of “service with a smile.”

## Data Availability Statement

The raw data supporting the conclusions of this article will be made available by the authors, without undue reservation.

## Ethics Statement

Ethical review and approval was not required for the study on human participants in accordance with the local legislation and institutional requirements. The patients/participants provided their written informed consent to participate in this study.

## Author Contributions

XZ made contributions to conceptualization, data collection, and drafting the article. NF made contributions to conceptualization, data analysis, and drafting of the article. YF and PF made contributions to conceptualization and drafting of the article. All authors contributed to the article and approved the submitted version.

## Conflict of Interest

The authors declare that the research was conducted in the absence of any commercial or financial relationships that could be construed as a potential conflict of interest.
